# Evaluation of a system for sorbent‐assisted peritoneal dialysis in a uremic pig model

**DOI:** 10.14814/phy2.14593

**Published:** 2020-12-05

**Authors:** Maaike K. van Gelder, Joost C. de Vries, Frank Simonis, Anneke S. Monninkhof, Diënty H. M. Hazenbrink, Giulia Ligabue, Silvia Giovanella, Jaap A. Joles, Marianne C. Verhaar, Maria A. Bajo Rubio, Rafael Selgas, Gianni Cappelli, Karin G. F. Gerritsen

**Affiliations:** ^1^ Department of Nephrology and Hypertension University Medical Center Utrecht Utrecht The Netherlands; ^2^ Nanodialysis BV Oirschot The Netherlands; ^3^ Surgical, Medical, Dental, Morphology Sciences, Transplant, Oncology and Regenerative Medicine Department Division of Nephrology University of Modena and Reggio Emilia Modena Italy; ^4^ Nephrology Service Hospital Universitario La Paz. Institute for Health Research (IdiPAZ) IRSIN REDinREN Madrid Spain

**Keywords:** artificial, chronic, continuous flow peritoneal dialysis, kidney failure, kidneys, peritoneal dialysis, sorbent

## Abstract

A system for sorbent‐assisted peritoneal dialysis (SAPD) has been developed that continuously recirculates dialysate *via* a tidal mode using a single‐lumen peritoneal catheter with the regeneration of spent dialysate by means of sorbents. SAPD treatment may improve plasma clearance by the maintenance of a high plasma‐to‐dialysate concentration gradient and by increasing the mass transfer area coefficient (MTAC) of solutes. The system is designed for daily 8‐hr treatment (12 kg, nighttime system). A wearable system (2.3 kg, daytime system) may further enhance the clearance of phosphate and organic waste solutes during the day. Uremic pigs (*n* = 3) were treated with the day‐ (*n* = 3) and nighttime system (*n* = 15) for 4–8 hr per treatment. Plasma clearance (Cl), MTAC, and total mass transport (MT) of urea, creatinine, phosphate, and potassium were compared with a static dwell (*n* = 28). Cl, MTAC, and MT of urea, creatinine, phosphate, and potassium were low in the pig as compared to humans due to the pig's low peritoneal transport status and could be enhanced only to a limited extent by SAPD treatment compared with a static dwell (nighttime system: Cl urea: ×1.5 (*p* = .029), Cl creatinine: ×1.7 (*p* = .054), Cl phosphate: ×1.5 (*p* = .158), Cl potassium: ×1.6 (*p* = .011); daytime system: Cl creatinine: ×2.7 (*p* = .040), Cl phosphate: ×2.2 (*p* = .039)). Sorbent‐assisted peritoneal dialysis treatment in a uremic pig model is safe and enhances small solute clearance as compared to a static dwell. Future studies in humans or animal species with higher peritoneal transport should elucidate whether our SAPD system enhances clearance to a clinically relevant extent as compared to conventional PD.

## Graphical abstract

A system for sorbent‐assisted peritoneal dialysis (SAPD) has been developed that continuously recirculates dialysate via a tidal mode using a single lumen peritoneal catheter with regeneration of spent dialysate by means of sorbents. SAPD treatment may improve plasma clearance by maintenance of a high plasma‐to‐dialysate concentration gradient and by increasing the mass transfer area coefficient (MTAC) of solutes. The system is designed for daily 8‐hr treatment (12 kg, nighttime system). A wearable system (2.3 kg, daytime system) may further enhance clearance of phosphate and organic waste solutes during the day.

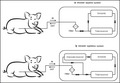

## 1 INTRODUCTION

Peritoneal dialysis (PD) is a life‐sustaining renal replacement therapy for patients with end‐stage kidney disease (ESKD). Although the vast majority (88%) of dialysis patients is treated with hemodialysis (HD) (Fresenius Medical Care, 2018), PD has several advantages compared with HD. Peritoneal dialysis is a blood‐free technique that uses relatively simple access to the abdomen and can be performed in the home environment. It provides more continuous and gradual removal of excess water and waste solutes. However, PD has several disadvantages. Clearance is relatively low compared with HD because diffusive solute transport rapidly decreases during a dwell due to the disappearance of the plasma‐to‐dialysate concentration gradient across the peritoneal membrane (Bammens, Evenepoel, Verbeke, & Vanrenterghem, 2003; Eknoyan et al., 2002). In addition, technique survival is limited to a median of 3.7 years (Mujais & Story, 2006) after which patients have to switch to the more invasive and expensive HD treatment (Aguirre & Abensur, 2011). Main causes of technique failure are recurrent peritonitis (Davenport, 2009) and membrane failure (Wu et al., 2012). Both recurrent peritonitis and exposure of the peritoneal membrane to very high initial glucose concentrations needed for osmotic fluid removal cause pathological changes of the peritoneal membrane (e.g., neoangiogenesis and fibrosis) (Wu et al., 2012) and may eventually lead to ultrafiltration failure (Aguirre & Abensur, 2011).

**FIGURE 1:**
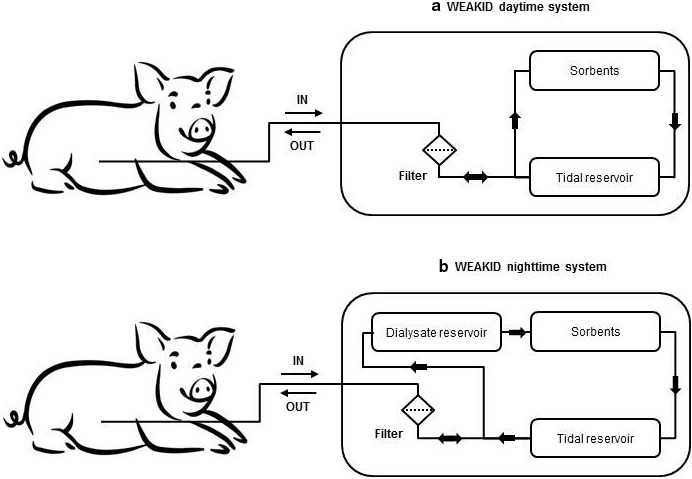
Schematic representation of the SAPD day‐ (A) and nighttime system (B). Peritoneal effluent is continuously recirculated via a tidal mode into and out of the SAPD system. With the nighttime system, peritoneal effluent first passes the 9‐L dialysate reservoir, then the sorbents, after which dialysate is temporarily stored in the tidal reservoir until the outflow phase starts and dialysate can be returned into the peritoneal cavity. A filter is placed between the sorbents and the peritoneal cavity to prevent particulate matter from entering the peritoneal cavity. IN, ingoing line (i.e., dialysate flows into the device); OUT, outgoing line (i.e., dialysate flows out of the device). Pig by Alice Noir from the Noun Project

Recently, we demonstrated the safety and efficacy of a system for sorbent‐assisted peritoneal dialysis (SAPD) in vitro (Gelder, Simonis, & Monninkof, 2019). The SAPD system comprises a wearable sorbent‐based device (Figure 1a “the SAPD daytime system,” weight: 2.3 kg) that is combined with a dialysate reservoir (provided in a trolley) during the night (Figure 1b “the SAPD nighttime system,” weight: 12 kg) to allow for sufficient urea and potassium removal. The SAPD nighttime system is intended to be used daily for 8 hr per night. Optionally, the wearable SAPD system can be used during the day for additional removal of phosphate and non‐urea organic waste solutes. The SAPD system aims to improve uremic toxin removal and prolong technique survival compared with conventional PD by introducing the following innovations: first, instead of performing 4–6 exchanges per day, only one filling per day is used with SAPD treatment that is continuously recirculated and regenerated by sorbent technology. The continuous flow of fresh dialysate along the peritoneal membrane is expected to enhance solute removal due to an increase of the mass transfer area coefficient (MTAC), as observed with continuous flow‐through peritoneal dialysis (CFPD) (Cruz et al., 2001; Diaz‐Buxo, Cruz, & Gotch, 2000; Freida & Issad, 2003; Raaijmakers, Schroder, & Gajjar, 2011; Shinaberger, Shear, & Barry, 1965), and maintenance of a high plasma‐to‐dialysate concentration gradient. Second, the SAPD system continuously releases glucose at a slow and controlled rate, maintaining a relatively constant intraperitoneal glucose concentration that is expected to induce osmotic fluid removal at a constant rate. In this way, very high initial glucose concentrations, as used during conventional PD to maintain an osmotic gradient and net fluid removal at the end of a dwell, are no longer required. Third, by reducing the number of (dis)connections of the peritoneal catheter, SAPD treatment may lower peritonitis rates (Fijter et al., 1991).

The aim of the present study was to assess the safety and compare the efficacy of the SAPD system in respect to a conventional static dwell (i.e., standard peritoneal permeability analysis, SPA) in terms of the MTAC and plasma clearance of uremic toxins, and ultrafiltration rate (UFR) in a uremic pig model. In addition, acute systemic toxicity was evaluated by assessing general clinical condition, electrolyte balance, and acid‐base balance during treatment.

## 2 METHODS

### 2.1 Experimental animal

Three female Yorkshire pigs (Sus scrofa domesticus), weighing 45–130 kg (bodyweight increased during the study) were used, since pigs have a similar bodyweight (and distribution volume), anatomy and physiology to humans and have already been successfully used for the evaluation of PD (Roberts & Zaragosa, 2016). Uremia was established by subtotal renal artery embolization. Gentamicin (10 mg/kg 2dd for 7 days) was administered prior to the testing of the SAPD system to induce acute‐on‐chronic kidney injury, resulting in plasma concentrations of uremic toxins in the range of those observed in dialysis patients. Peritoneal access was established with a straight Tenckhoff PD catheter (Argyle, Covidien, Tenckhoff, 1 cuff, 47 cm).

### 2.2 Materials

A schematic presentation of the SAPD day‐ and nighttime systems is presented in Figure 1. The sorbent cartridge comprises an anion‐binder (ferric oxide hydroxide, FeOOH) for the removal of phosphate and activated carbon for the removal of organic waste solutes (e.g., creatinine). The sorbents were equilibrated at [Na^+^] 132 mmol/L, [Cl^−^] 97 mmol/L, [lactate] 10 mmol/L and [HCO_3_^‐^] 25 mmol/L at pH 7.0. The dialysate reservoir contained Physioneal 35 (Baxter, GmBH Germany) glucose 1.36% (*n* = 7), 1.72% (*n* = 5) or 2.27% (*n* = 3) for comparison of UFR.

### 2.3 Experimental procedures

Three 8‐hr experiments with the daytime system (*n* = 3 experiments in *n* = 1 pig, Table 1), fifteen 4–8‐hr experiments with the nighttime system (*n* = 7, *n* = 5 and *n* = 3 per pig, Table 2) and 28 SPAs (*n* = 16, *n* = 8 and *n* = 4 per pig), were performed in these three pigs for comparison of plasma clearance, MTAC, and total mass transport between SAPD and SPA (i.e., static dwell) experiments.

**TABLE 1:** Experimental conditions during the testing of the SAPD daytime system

Experiment	Treatment time (h)	Tidal volume (mL)	Flow IN (mL/min)	Flow OUT (mL/min)	Qd (mL/min)	Peritonitis
1	8	200	100	200	67	YES
2	8	300	100	200	67	YES
3	8	300	100	100	50	YES
Mean ± *SD*			100 ± 0	167 ± 47	61 ± 8	

Note

Flow IN, dialysate flow rate into the SAPD system; Flow OUT, dialysate flow rate out of the SAPD system; Qd, mean effective dialysate flow rate.

**TABLE 2:** Experimental conditions during the testing of the SAPD nighttime system

Experiment	Treatment time (h)	Tidal volume (mL)	Flow IN (mL/min)	Flow OUT (mL/min)	Qd (mL/min)	[Glucose] dialysate reservoir (%)^a^	Peritonitis
1	4	300	75	150	50	1.36	YES
2	4	380	150	190	84	1.36	YES
3	6	440	95	190	63	1.36	NO
4	8	300	200	200	100	1.36	YES
5	8	310	165	185	88	1.36	YES
6	8	340	165	170	84	1.36	NO
7	8	340	165	170	84	1.36	NO
8	8	250	160	200	89	1.72	YES
9	8	250	150	190	84	1.72	NO
10	8	250	150	190	84	1.72	YES
11	8	350	155	150	76	1.72	YES
12	8	530	170	160	82	1.72	YES
13	8	280	155	165	81	2.27	NO
14	8	300	155	180	84	2.27	YES
15	8	380	150	190	84	2.27	YES
Mean ± *SD*			151 ± 29	179 ± 16	81 ± 11		

Note

Abbreviations: Flow IN, dialysate flow rate into the SAPD system; Flow OUT, dialysate flow rate out of the SAPD system; Qd, mean effective dialysate flow rate.

^a^ Glucose concentration in the dialysate reservoir at the start of treatment. *n* = 15 experiments were performed in *n* = 3 pigs (*n* = 7, *n* = 5, and *n* = 3 per pig).

One day prior to SAPD experiments, 2 L of Physioneal 35 glucose 1.36% (Baxter) or Extraneal 7.5% (Baxter) were instilled in the peritoneal cavity to allow for the equilibration of blood and PD fluid. A minimum intraperitoneal volume of 2 L was required in the pig to achieve a mean effective dialysate flow rate (Qd, Formula 1) > 50 ml/min (i.e., the minimum intended flow rate in patients). Prior to start, leukocyte and neutrophil count were measured in the overnight dwell and a sample was taken for microbiological culture. At the start and end of experiments with the SAPD nighttime system, 1 L of fresh PD fluid was instilled to determine intraperitoneal volume based on total protein concentration in peritoneal effluent before and after the installation of fresh PD fluid (Formula 11). Next, the SAPD system was connected to the pig's peritoneal catheter and after the start of the dialysate pump, peritoneal effluent was continuously recirculated through the SAPD system *via* a tidal mode, that is, alternate in‐ and efflux of dialysate into‐ and out of the SAPD system, respectively. When catheter outflow obstruction occurred, the pig was sedated with propofol so that it could be repositioned to resolve the outflow obstruction.

Venous blood samples were taken from a jugular line before start and after 2, 4, 6, and 8 hr of treatment for measurement of urea, creatinine, phosphate, potassium, sodium, chloride, bicarbonate, calcium, magnesium, albumin, glucose, lactate dehydrogenase, free hemoglobin, hemoglobin, leukocyte, and thrombocyte counts and venous blood gas. Peritoneal effluent was collected before start and dialysate samples were taken from the in‐ and outgoing line (when dialysate flows into and out of the SAPD system, respectively) after 10 and 30 min, and 1, 2, 3, 4, 6, and 8 hr of treatment for measurement of urea, creatinine, phosphate, potassium, sodium, chloride, bicarbonate, lactate, calcium, magnesium, glucose, osmolality and pH. Thirteen SAPD experiments were performed, while the pigs had peritonitis (unintentional). Because peritonitis caused a considerable increase in transport status (and MTAC), control experiments were also performed during peritonitis (*n* = 10 SPAs during peritonitis, *n* = 18 SPAs without peritonitis) for comparison.

A veterinarian and experienced biotechnician were present during the experiment to monitor the pig's clinical conditions.

### 2.4 Standard peritoneal permeability analysis

The standard peritoneal permeability analysis (SPA) is a tool to assess peritoneal membrane permeability characteristics which uses a 4‐hr dwell with Physioneal 35 1.36% glucose. One day prior to the SPA, 2 L of Physioneal 35 glucose 1.36% or Extraneal 7.5% (Baxter) were instilled in the peritoneal cavity, similar to SAPD experiments. The next day, a complete drain of the overnight dwell was performed and a sample was taken from the drain bag for measurement of urea, creatinine, phosphate, glucose, total protein, leukocyte, and neutrophil count. In addition, a sample was taken for microbial culture. Subsequently, 2 L of Physioneal 35 1.36% glucose (*n* = 26) or 2.27% glucose (*n* = 2) were instilled and PD effluent samples were collected at 0, 10, 20, 30, 60, 120, 180, and 240 min for measurement of urea, creatinine, phosphate, glucose and blue dextran. At each time point, 100–200 ml of PD effluent was drained into a sterile empty dialysate bag, a sample was collected, and the remaining volume was re‐instilled. After 240 min, a complete drain was performed, followed by rinsing of the peritoneal cavity with 1 L of fresh Physioneal 35 (1.36% glucose). Total protein concentration in the overnight dwell and in the *t* = 0 min sample was used to calculate residual volume at the start of the SPA, and total protein concentration in the *t* = 240 min sample and in the drain after flushing at the end of the SPA were used to calculate residual volume after the complete drain at the end of the SPA (Formula 11). Venous blood samples were taken at 0, 120, and 240 min for measurement of urea, creatinine, and phosphate concentrations.

### 2.5 Laboratory measurements

All clinical biochemistry and hematological measurements were performed at the hospital laboratory of the UMC Utrecht. Urea, creatinine, potassium, phosphate, sodium, calcium, magnesium, bicarbonate, lactate, osmolality, albumin, and LDH concentrations were analyzed with a routine chemistry analyzer (DxAU 5811, Beckman Coulter). Hemoglobin, leukocyte, and thrombocyte concentrations were analyzed with a routine hematology analyzer (CELL‐DYN Sapphire, Abbott Diagnostics). Free hemoglobin was measured using dual‐wavelength spectrophotometry. Venous blood gasses were analyzed using a blood gas analyzer (RAPIDLab type 1265; Siemens Medical Solutions Diagnostics B.V.).

### 2.6 Calculations and statistics

The mean effective dialysate flow rate during SAPD treatment was calculated as follows:

(1) $$Qd=\frac{Q_{\text{IN}}\times t_{\text{IN}}}{t_{\text{IN}}+t_{\text{OUT}}}$$

where *Q*d = mean effective dialysate flow rate, *Q*_IN_ = dialysate flow rate into the system, *t*_IN_ = time of the inflow phase, and *t*_OUT_ = time of the outflow phase. *t*_IN_ and *t*_OUT_ were calculated by dividing the tidal volume by *Q*_IN_ and *Q*_OUT_, respectively.

Peritoneal catheter flow recirculation occurs when dialysate returning from the SAPD system into the peritoneal cavity re‐enters the peritoneal catheter when the tidal flow changes direction from out‐ to ingoing. The degree of peritoneal catheter flow recirculation (%) was calculated based on the creatinine concentration in the peritoneal effluent sample at *t* = 8 hr and in the complete drain after treatment.

(2) $$\text{CR}=\frac{{\left[\text{creat}\right]}_{t2}‐{\left[\text{creat}\right]}_{t1}}{{\left[\text{creat}\right]}_{t2}}\times 100\%$$

where CR = catheter flow recirculation (%), [creat]*_t_*_1_ = creatinine concentration in PD effluent at *t* = 8 hr, and [creat]*_t_*_2_ = creatinine concentration in the complete drain.

Cumulative removal (or release) by the SAPD system from dialysate and plasma clearance was calculated using the following formulas:

(3) $$A_{\left(t1\to t2\right)}=\frac{\left(D_{\text{IN}}‐D_{\text{OUT}}\right)t1+\left(D_{\text{IN}}‐D_{\text{OUT}}\right)t2}{2}\times Od\times t$$

where *A*_(_ *_t_*_1→_ *_t_*_2)_ = amount removed by the SAPD system between *t*1 and *t*2, *D*_IN_ = dialysate concentration in the ingoing line (i.e., upstream of the 10‐L reservoir and/or sorbent cartridge), *D*_OUT_ = dialysate concentration in the outgoing line (downstream of the sorbent cartridge), *Q*d = mean effective dialysate flow rate (L/min) and *t* = time (min) between two consecutive measurements (*t*2−*t*1).

(4) $$Cl=\frac{MT_{\text{total}}/t}{P}$$

where *Cl* = plasma clearance (mL/min), *MT*_total_ = total mass transport, *t* = time (min) between two consecutive measurements, and *P* = mean plasma concentration calculated as the mean of five measurements at *t* = 0, 2, 4, 6, and 8 hr of SAPD treatment.

Total mass transport of solutes (removal or release) from (or into) the pig during SAPD experiments was calculated as the sum of the cumulative removal (or release) by the SAPD system, the amount removed by sampling, and the amount present in the intraperitoneal volume at the end of treatment, minus the amount present in the intraperitoneal volume at the start of the dwell.

(5) $$MT_{\text{total}}=A+A_{\text{samples}}+\left({\text{IPV}}_{t2}\times D_{t2}\right)‐\left({\text{IPV}}_{t1}\times D_{t1}\right)$$

where *MT*_total_ = total mass transport (removal or release) from (or into) the pig, *A* = amount removed (or released) by the SAPD system, *A*_samples_ = amount removed by sampling, D*_t_*_1_ = dialysate concentration at *t*1, D*_t_*_2_ = dialysate concentration at *t*2, IPV*_t_*_1_ = intraperitoneal volume at *t*1 (sum of the filling volume and residual volume prior to filling), and IPV*_t_*_2_ = intraperitoneal volume at the end of treatment (sum of the drained volume and residual volume after drainage).

Convective mass transport of waste solutes was calculated based on the sieving coefficient, UFR, and the mean solute concentration in the peritoneal membrane as described by Waniewski, Werynski, Heimburger, and Lindholm (1991).

(6) $$MT\text{convective}=\text{SC}\times C_{\text{mean}}\times t$$

where MTconvective = convective mass transport, SC = sieving coefficient, *C*_mean_ = mean concentration in the peritoneal membrane, and *t* = time between two consecutive measurements.

(7) $$C_{\text{mean}}=\left(\left(1‐F\right)\times P\right)+\left(F\times D\right)$$

where *C*_mean_ = mean solute concentration in the peritoneal membrane, *F* = weighing factor (0.5 for small water soluble solutes), P = plasma concentration, and D = dialysate concentration.

Diffusive mass transport (Formula 8) was calculated by subtracting convective mass transport (Formula 6) from total mass transport (Formula 5) as described by Waniewski et al. (1991).

(8) $$MT_{\text{diffusive}}=MT_{\text{total}}‐MT_{\text{convective}}$$

where *MT*_diffusive_ = diffusive mass transport, *MT*_total_ = total mass transport, *MT*_convective_ = convective mass transport.

The MTAC during SAPD experiments was calculated by dividing the diffusive mass transport by the time interval and plasma‐to‐dialysate concentration gradient.

(9) $$MTAC\left(t1\to t2\right)=\frac{MT_{\text{diffusive}}\left(t1\to t2\right)/t\;}{\left(Pt1‐Dt1+Pt2‐Dt2\right)/2}$$

where *MTAC* = mass transfer area coefficient (mL/min), *MT*_diffusive_ = diffusive mass transport, *P_t_*_1_ = plasma concentration at *t*1, *P_t_*_2_ = plasma concentration at *t*2, D*_t_*_1_ = dialysate concentration at *t*1, D*_t_*_2_ = dialysate concentration at *t*2, and *t* = time between two consecutive measurements (*t*1−*t*2).

UFR during SAPD and SPA experiments was calculated as follows:

(10) $$UFR=\frac{\left(RV_{\mathrm{tx}}+V_{\mathrm{tx}}+V_{\text{samples}}\right)‐\left(RV_{t0}+V_{t0}\right)}{t}$$

where *UFR* = ultrafiltration rate, *RV_t_*_0_ = residual intraperitoneal volume before the installation of fresh PD solution, *RV_tx_* = residual intraperitoneal volume after the complete drain, *V_t_*_0_ = volume of the PD solution bag, *V_tx_* = volume of the complete drain bag, *V*_samples_ = total sample volume, and *t* = dwell time.

Of note, the effective lymphatic adsorption rate (ELAR) could not be quantified because no reliable intraperitoneal volume marker was available for monitoring the intraperitoneal volume during SPA and SAPD experiments, as dextran 70 was not available, use of blue dextran gave unreliable results, and albumin stuck to the dialysis membrane of the SAPD system. UFR was, therefore, estimated based on the intraperitoneal volume at the start and end of the experiment, corrected for total sampling volume.

The residual volume before and after SAPD and SPA experiments was calculated based on total protein concentration in the peritoneal effluent before and after the installation of fresh PD fluid.

(11) $$RV=\frac{Vi\times {\left[\text{total protein}\right]}_{t2}}{{\left[\text{total protein}\right]}_{t1}‐{\left[\text{total protein}\right]}_{t2}}$$

where *RV* = residual intraperitoneal volume, *Vi* = volume of the instilled (fresh) PD fluid, [total protein]*_t_*_1_ = total protein concentration in PD effluent before rinsing, and [total protein]*_t_*_2_ = total protein concentration in PD effluent after rinsing.

Total mass transport and plasma clearance during SPA experiments were calculated using the following formulas:

(12) $$MT_{\text{total}}=A_{\text{samples}}+\left(IPV_{t240}\times D_{t240}\right)‐\left(RV_{t0}\times D_{t0}\right)$$

where *MT*_total_ = total mass transport (removal or release) from (or into) the pig, *A*_samples_ = amount removed by sampling, D*_t_*_0_ = dialysate concentration at *t*0, D*_t_*_240_ = dialysate concentration at *t* = 240 min, *IPV_t_*_0_ = intraperitoneal volume at *t*0 (sum of the filling volume and residual volume prior to filling), and *IPV_t_*_240_ = intraperitoneal volume at *t* = 240 min (sum of the drained volume and residual volume after drainage).

(13) $$Cl=\frac{MT_{\text{total}}/t}{P}$$

where *Cl* = plasma clearance (mL/min), *MT*_total_ = total mass transport (removal or release) from (or into) the pig, *t* = 240 min (= dwell time), and *P* = mean plasma concentration calculated as the mean of two measurements before and after the SPA.

MTAC urea, creatinine, and phosphate during SPA experiments, were calculated according to Waniewski *et al*. (Krediet, Lindholm, & Rippe, 2000):

(14) $$MTAC=\frac{V_{m}}{t}\times \ln\left[\frac{V_{t0}^{\left(1‐F\right)}\left(P‐D_{t0}\right)}{V_{t240}^{\left(1‐F\right)}\left(P‐D_{t240}\right)}\right]$$

where *MTAC* = mass transfer area coefficient, *V_m_* = mean intraperitoneal volume, *t* = dwell time, *V_t_*_0_ = intraperitoneal volume at *t* = 0 min calculated based on total protein concentration in the peritoneal effluent before and after the installation of fresh PD fluid, *V_t_*_240_ = intraperitoneal volume at *t* = 240 min, D*_t_*_0_ = dialysate concentration at *t* = 0 min, D*_t_*_240_ = dialysate concentration at *t* = 240 min, *P* = mean plasma concentration calculated as the mean of two measurements before and after the SPA, and *F* = weighing factor which estimates the relative importance of convective transport in comparison to diffusive transport (*F* = 0.5 for small, freely diffusible solutes such as urea and creatinine) (Waniewski et al., 1991).

Mean electrolyte concentrations, osmolality, and pH in the in‐ and outgoing line of the SAPD system were calculated as a time‐weighted mean of nine measurements (*t* = 0, 10, 60, 120, 180, 240, 300, 360, and 480 min) during treatment.

A Student's paired *t*‐test was used to compare laboratory measurement before and after treatment and for comparison of consecutive SAPD and SPA experiments using GraphPad Prism Software (version 8.00; La Jolla, California, USA). The mean plasma clearance and MTAC per 8‐hr SAPD‐session were compared with plasma clearance and MTAC during 4‐hr SPA experiments. Total mass transport during the 8‐hr SAPD session was divided by two for comparison with a 4‐hr SPA. To study the association between dialysate flow rate, tidal volume, intraperitoneal volume, bodyweight, and the MTAC and plasma clearance of urea, creatinine, and phosphate, univariate linear regression was performed using SPSS (version 18; SPSS Inc. Headquarters, Chicago, IL, USA). For non‐normally distributed data, log transformation was applied to allow for parametric analysis. Results are expressed as beta (β) regression coefficients with *p‐*value. In all statistical testing, a two‐tailed *p* < .05 was considered statistically significant.

## 3 RESULTS

### 3.1 Safety analysis

#### 3.1.1 Treatment tolerance

No signs of abdominal pain or animal discomfort were observed with dialysate flow rates up to 200 ml/min into‐ and out of the peritoneal cavity for 8 hr. No serious adverse events occurred that were associated with SAPD treatment.

#### 3.1.2 Blood measurements

Blood measurements at the start and end of treatment with the day‐ and nighttime experiments are presented in Table 3. Mean electrolyte and glucose concentrations, osmolality, and pH in the in‐ and outgoing line of the SAPD system are presented in Table 4.

**TABLE 3:** Blood measurements at the start and end of the SAPD day‐ and nighttime experiments (8 hr per experiment)

Measurement	Nighttime system (*n* = 15)			Daytime system (*n* = 3)		
	Start	End	*P* *	Start	End	*P* *
Sodium (mmol/L)	141 ± 0.9	138 ± 3.1	0.189	140 ± 2.2	139 ± 2.6	0.228
Potassium (mmol/L)^a^	5.2 ± 0.1	4.8 ± 0.5	0.278^a^	4.1 ± 0.5	3.9 ± 0.5	0.063
Chloride (mmol/L)	106 ± 0.8	100 ± 0.5	**0.023**	100 ± 5.3	98 ± 4.8	**0.009**
Calcium (mmol/L)	2.78 ± 0.07	2.58 ± 0.00	0.063	2.61 ± 0.31	2.53 ± 0.14	0.320
Magnesium (mmol/L)	1.20 ± 0.05	1.05 ± 0.07	**0.033**	1.41 ± 0.36	1.26 ± 0.40	0.057
Bicarbonate (mmol/L)	29.1 ± 1.6	31.4 ± 2.9	0.149	31.3 ± 5.2	31.8 ± 3.8	0.374
Phosphate (mmol/L)	2.40 ± 0.16	2.10 ± 0.22	**0.017**	2.25 ± 0.31	2.22 ± 0.35	0.325
Urea (mmol/L)^a^	4.6 ± 2.6	3.9 ± 1.8	0.426^a^	13.1 ± 6.7	11.8 ± 5.8	**0.011**
Creatinine (µmol/L)	441 ± 145	386 ± 116	0.120	746 ± 425	732 ± 427	0.404
Albumin (g/L)	24.5 ± 0.9	23.9 ± 1.1	0.096	26.1 ± 7.0	28.3 ± 2.3	0.174
Glucose (mmol/L)	5.8 ± 0.1	6.9 ± 0.3	**0.034**	5.8 ± 0.9	5.9 ± 0.6	0.928
Lactate dehydrogenase (U/L)	813 ± 79	779 ± 81	0.485	766 ± 362	740 ± 386	0.521
Free hemoglobin (mg/L)	22 ± 11	21 ± 7	0.982	18 ± 10	20 ± 10	0.073
Hemoglobin (mmol/L)	5.4 ± 0.4	6.9 ± 0.3	0.093	5.6 ± 0.5	5.5 ± 0.5	0.220
Thrombocytes (×10^9/L)	310 ± 189	444 ± 87	0.457	383 ± 196	380 ± 176	0.155
Leukocytes (×10^9/L)	13.6 ± 3.9	17.9 ± 3.2	0.423	12.0 ± 3.0	13.4 ± 4.2	0.263
Venous pH	7.39 ± 0.03	7.46 ± 0.01	0.078	7.42 ± 0.06	7.44 ± 0.06	**0.037**

Note

Data are presented as mean ± standard deviation.

^a^ The SAPD daytime system does not comprise a urea removal system or cation exchanger for the removal of potassium. *n* = 15 experiments were performed with the nighttime system in *n* = 3 pigs (*n* = 7, *n* = 5, and *n* = 3 per pig) and *n* = 3 experiments were performed with the daytime system in *n* = 1 pig.

* Statistical significance between end and start was determined using a Student's paired *t*‐test. Significant differences are shown in bold font.

**TABLE 4:** Mean electrolyte concentrations, osmolality, and pH in the SAPD in‐ (IN) and outgoing (OUT) line during experiments with the SAPD day‐ and nighttime systems

Measurement	Daytime system (*n* = 3)			Nighttime system (*n* = 15)			Commercial PD fluids‡
	IN	OUT	*P* *	IN	OUT	*P* *	
Potassium (mmol/L)^a^	4.2 ± 0.4	4.1 ± 0.4	**<0.001**	2.2 ± 0.3	1.6 ± 0.3	**<0.001**	0
Phosphate (mmol/L)	0.79 ± 0.09	0.57 ± 0.11	**0.020**	0.30 ± 0.18	0.12 ± 0.08	**<0.001**	0
Urea (mmol/L)^a^	3.3 ± 1.4	3.1 ± 1.4	0.057	4.3 ± 2.0	3.1 ± 1.5	**<0.001**	0
Creatinine (µmol/L)	128 ± 22	52 ± 12	**0.029**	103 ± 73	39 ± 32	**<0.001**	0
Sodium (mmol/L)	128 ± 2.9	129 ± 2.9	0.109	127 ± 4.8	128 ± 4.1	**0.005**	132–134
Chloride (mmol/L)	99 ± 1.0	99 ± 1.2	0.691	98 ± 2.7	98 ± 2.4	0.091	95–105
Bicarbonate (mmol/L)	26.9 ± 0.7	27.2 ± 0.9	0.053	25.8 ± 2.1	25.7 ± 1.6	0.394	0–34
Lactate (mmol/L)	6.2 ± 2.9	6.5 ± 2.8	**0.020**	8.2 ± 1.6	8.9 ± 1.6	**<0.001**	0–40
Calcium (mmol/L)	1.25 ± 0.04	1.16 ± 0.05	**<0.001**	1.34 ± 0.15	1.35 ± 0.15	0.253	1.25–1.75
Magnesium (mmol/L)	0.46 ± 0.02	0.43 ± 0.01	0.184	0.35 ± 0.11	0.30 ± 0.07	**0.004**	0.25–0.50
Osmolality (mOsmol/kg)	304 ± 3.1	296 ± 3.2	**0.024**	316 ± 10	311 ± 0.0	0.145	284–483
pH	7.7 ± 0.0	7.8 ± 0.0	0.057	7.6 ± 0.1	7.6 ± 0.1	0.676	5.5–7.4

Note

Data are presented as mean ± standard deviation.

^a^ The SAPD daytime system does not comprise a urea removal system or cation exchanger for the removal of potassium. ‡Commercial PD fluids include Balance® (Fresenius Medical Care, FMC), BicaVera® (FMC), Gambrosol® Trio (FMC), Physioneal® (Baxter), Extraneal® (Baxter), and Nutrineal® (Baxter). *n* = 15 experiments were performed with the nighttime system in *n* = 3 pigs (*n* = 7, *n* = 5, and *n* = 3 per pig) and *n* = 3 experiments were performed with the daytime system in *n* = 1 pig.

* Statistical significance between OUT and IN was determined using a Student's paired *t*‐test. Significant differences are shown in bold font.

With the daytime system, plasma calcium concentrations tended to decrease as a result of calcium removal by the sorbents (2.9 ± 0.6 mmol). Plasma calcium concentrations were stable with the nighttime system despite limited calcium release (0.34 ± 0.83 mmol) from the dialysate reservoir that contained [Ca^2+^] 1.75 mmol/L. Plasma magnesium concentrations decreased with the day‐ and nighttime systems due to removal by the sorbents (1.16 ± 0.28 mmol and 1.74 ± 0.82 mmol, respectively) and the relatively low magnesium concentration (0.25 mmol/L) in the dialysate reservoir (Physioneal 35). Venous pH increased with the day‐ and nighttime systems due to the combination of chloride removal (45.6 ± 8.6 mmol and 9.8 ± 60.5 mmol, respectively) and (limited) base release (Table 6).

### 3.2 Efficacy analysis

#### 3.2.1 Effect of SAPD on plasma clearance, MTAC and solute removal

Treatment with the day‐ and nighttime systems caused an increase in plasma clearance and MTAC compared with a static dwell (Table 5). A constant high plasma‐to‐dialysate concentration gradient was maintained during the 8‐hr SAPD treatment (Figure S1). The dialysate‐to‐plasma concentration ratios at 4 hr were lower during SAPD than during SPA experiments (Table S2). Of note, all SPA experiments showed a low peritoneal transport status in the pig. The dialysate‐to‐plasma concentration ratio for creatinine at 4 hr was 0.41 ± 0.10 in the absence of peritonitis (“low transporter”), compared with 0.78 (0.53–0.98) (“high‐average transporter”) in humans (Smit et al., 2000).

**TABLE 5:** Plasma clearance, mass transfer area coefficient (MTAC), and total mass transport of urea, creatinine, phosphate, and potassium during SAPD and SPA experiments

	Peritonitis	SPA (*n* = 28)	SAPD daytime (*n* = 3)	SAPD daytime versus SPA	*P* *	SAPD nighttime (*n* = 15)	SAPD nighttime versus SPA	*P* *
Plasma clearance (mL/min)								
Urea	NO	5.4 ± 1.6				6.8 ± 1.7	×1.3	0.051
	YES	5.6 ± 1.9	3.4 ± 1.4	×0.6	0.153^a^	8.6 ± 3.6	×1.5	**0.029**
Creatinine	NO	3.4 ± 0.7				3.9 ± 0.5	×1.2	0.161
	YES	4.0 ± 1.6	10.7 ± 2.0	×2.7	**0.040**	6.6 ± 3.9	×1.7	0.054
Phosphate	NO	2.7 ± 0.8				3.3 ± 0.8	×1.2	0.242
	YES	3.2 ± 1.5	7.2 ± 1.4	×2.2	0.053	4.9 ± 3.7	×1.5	0.158
Potassium	NO	7.4 ± 1.3				8.9 ± 1.0	×1.2	**0.023**
	YES	6.8 ± 1.1	2.9 ± 1.3	×0.4	**0.039** ^a^	10.9 ± 3.8	×1.6	**0.011**
MTAC (mL/min)								
Urea	NO	9.5 ± 2.1				11.7 ± 2.8	×1.2	**0.027**
	YES	13.5 ± 3.8	17.3 ± 6.9	×1.3	0.827^a^	15.2 ± 9.4	×1.1	0.296
Creatinine	NO	4.1 ± 1.2				5.0 ± 0.9	×1.2	0.050
	YES	7.3 ± 3.0	16.7 ± 3.3	×2.3	**<0.001**	9.1 ± 7.1	×1.3	**0.037**
Phosphate	NO	3.2 ± 1.1				3.9 ± 1.0	×1.2	0.077
	YES	5.6 ± 2.3	11.0 ± 2.1	×2.0	0.095	6.6 ± 6.2	×1.2	0.400
Potassium	NO	19.0 ± 2.7				18.6 ± 3.0	×1.0	0.928
	YES	18.8 ± 6.3	16.0 ± 4.1	×0.9	0.093^a^	22.8 ± 7.9	×1.2	0.241
MTtotal (mmol/ 4h)								
Urea^a^	NO	12.6 ± 6.2				21.5 ± 8.1	×1.7	**0.045**
	YES	16.9 ± 6.5	3.7 ± 2.3	×0.2	**0.015** ^a^	18.9 ± 10.8	×1.1	0.295
Creatinine	NO	0.5 ± 0.2				0.7 ± 0.4	×1.5	0.449
	YES	0.7 ± 0.4	1.1 ± 0.4	×1.5	0.240	0.8 ± 0.5	×1.2	0.125
Phosphate	NO	1.5 ± 0.3				1.7 ± 0.6	×1.1	0.373
	YES		4.0 ± 0.5	×2.1	**0.023**	2.2 ± 1.2	×1.2	0.302
Potassium^a^	NO	7.2 ± 1.6				8.2 ± 1.1	×1.1	0.170
	YES	6.5 ± 1.5	3.4 ± 1.4	×0.5	**0.044** ^a^	9.2 ± 3.7	×1.4	0.130

Note

MTAC, mass transfer area coefficient; MTtotal, total mass transport; SPA, standard peritoneal permeability analysis.

^a^ The SAPD daytime system does not comprise a urea removal system or cation exchanger for the removal of potassium. *n* = 15 experiments were performed with the nighttime system in *n* = 3 pigs (*n* = 7, *n* = 5, and *n* = 3 per pig), *n* = 3 experiments were performed with the daytime system in *n* = 1 pig and 28 SPA experiments were performed in *n* = 3 pigs (*n* = 8, *n* = 16, and *n* = 4 per pig).

* P‐value was calculated using a Student's paired *t*‐test for comparison of consecutive SAPD and SPA experiments.

#### 3.2.2 Determinants of the MTAC and clearance of urea, creatinine, and phosphate

Dialysate flow rates from the pig into the device of 75 to 200 ml/min (on average 151 ± 29 ml/min) and from the device to the pig of 150 to 200 ml/min (on average 179 ± 16 ml/min) were applied during experiments with the nighttime system, resulting in a mean effective dialysate flow rate of 81 ± 11 ml/min (Table 2). At higher flow rates catheter outflow obstruction occurred. Urea clearance related positively to mean effective dialysate flow rate (β 0.030, *p* < .001), but not to tidal volume, intraperitoneal volume or bodyweight. No association was observed between creatinine clearance, phosphate clearance, MTAC urea, MTAC creatinine, MTAC phosphate, and dialysate flow rate, bodyweight, tidal volume or intraperitoneal volume (Figure S2). Mean catheter flow recirculation was 31 ± 11% at a mean effective dialysate flow rate of 75 ± 14 ml/min.

#### 3.2.3 Ultrafiltration rate and glucose concentrations

**FIGURE 2:**
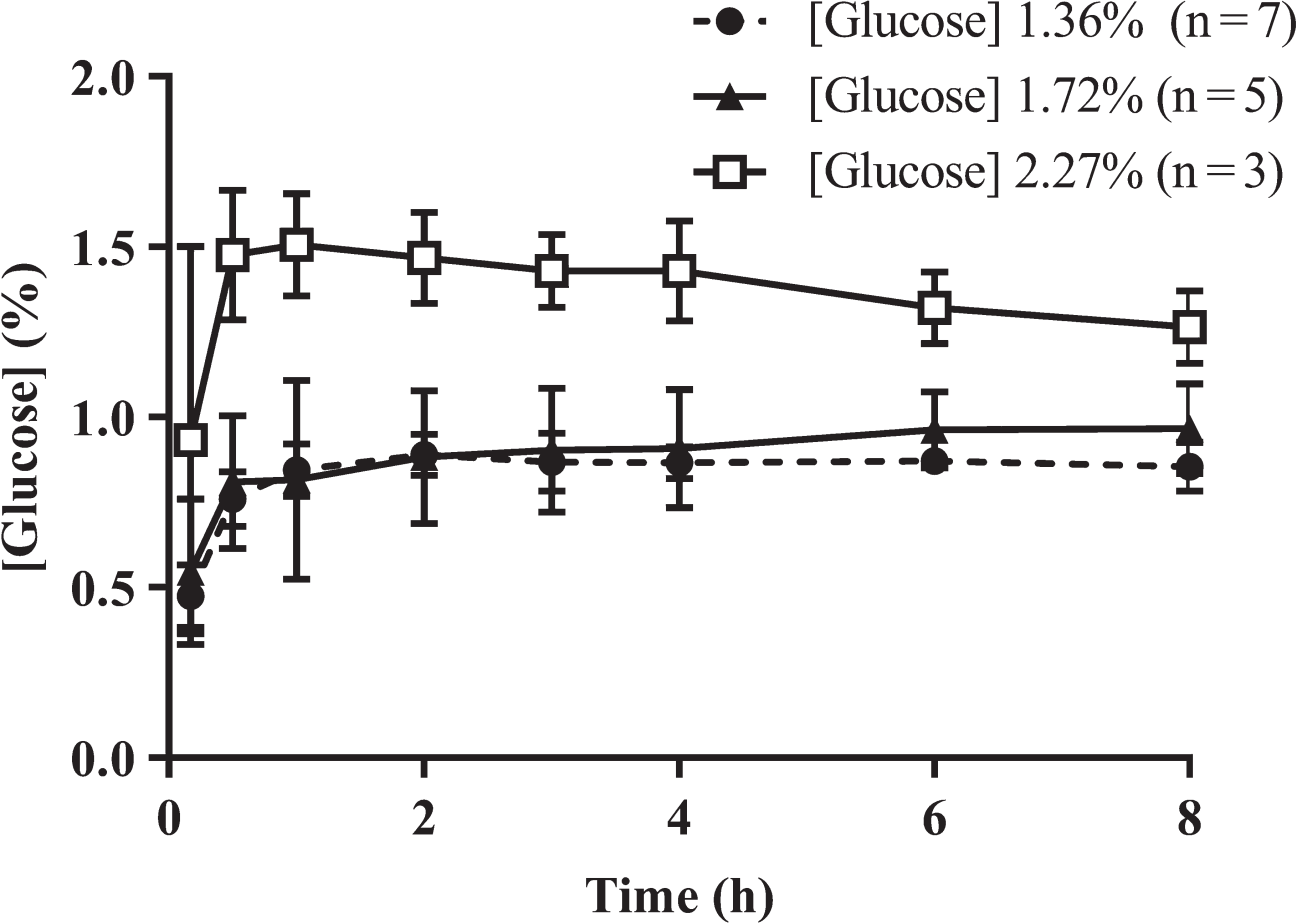
Dialysate glucose concentration (%) in the outgoing line of the SAPD nighttime system using Physioneal 35 with 1.36%, 1.72%, and 2.27% glucose in the 10‐L reservoir

Ultrafiltration rate during treatment with the SAPD nighttime system and the SPA experiments was very variable (Table S1) and the lymphatic absorption rate could not be determined. Therefore, although maximum intraperitoneal glucose concentrations were (not significantly) lower during SAPD experiments, no conclusion can be drawn with regard to the efficacy of ultrafiltration. Glucose was released by the SAPD system at a steady rate, maintaining a relatively constant glucose concentration in the dialysate that is returned into the peritoneal cavity (Figure 2).

#### 3.2.4 Bicarbonate and lactate release

Cumulative bicarbonate and lactate release into the peritoneal cavity was 7.8 ± 4.1 mmol and 9.3 ± 0.4 mmol, respectively, with the daytime system, resulting in a net buffer release of 17.1 mmol. With the nighttime system, cumulative bicarbonate removal was 9.3 ± 17.3 mmol and lactate release was 30.0 ± 19.2, resulting in net buffer release of 21 mmol (Table 4). Of note, the pig's plasma bicarbonate concentration at the start of SAPD treatment was high: 29.1 ± 1.6 mmol/L and 31.3 ± 5.2 mmol/L with the day‐ and nighttime systems (Table 6), respectively, limiting (daytime system) or preventing (nighttime system) bicarbonate release.

**TABLE 6:** Cumulative removal (or release) of bicarbonate, lactate, and glucose by the SAPD day‐ and nighttime systems

Measurement	Daytime system (*n* = 3)	Nighttime system (*n* = 3)
	A (mmol)	A (mmol)
Bicarbonate	−7.8 ± 4.1	9.3 ± 17.3
Lactate	−9.3 ± 0.4	−30.0 ± 19.2
Glucose 1.36%, Glucose 1.72%, Glucose 2.27%.	54 ± 14	−103 ± 55 −130 ± 64 −271 ± 70

Note

Positive and negative values represent removal and release, respectively. A, cumulative removal (or release) by the SAPD system. Glucose release when the dialysate reservoir contained glucose ^*^1.36%, †1.72%, and ‡2.27%.

## 4 DISCUSSION

In the present study, the safety and efficacy of the SAPD system, a novel miniature artificial kidney for continuous flow peritoneal dialysis, was evaluated in a uremic pig model. SAPD treatment was well tolerated and no serious adverse events occurred. Plasma clearance of small solutes during SAPD treatment increased compared with a static dwell. Depending on the solute, this could be partly attributed to a (limited) increase in MTAC. In addition, maintenance of a relatively high plasma‐to‐dialysate concentration gradient contributed to the enhanced clearance.

During SAPD treatment, dialysate is regenerated by means of sorbents. Ferric oxide hydroxide is used for the removal of anions (primarily phosphate) and activated carbon for the removal of organic waste solutes (e.g., creatinine). Since the affinity of activated carbon for urea is low (van Gelder, Jong, et al., 2020) and the system does not contain a cation exchanger, the nighttime system is equipped with a dialysate reservoir for (additional) removal of urea and potassium. During the experiments with the nighttime system, creatinine and phosphate were, indeed, primarily removed *via* binding to the sorbents (80 ± 7% and 75 ± 8% of total removal, respectively) and urea and potassium primarily by the dialysate reservoir (86 ± 11% and 97 ± 8% of total removal, respectively). However, total mass transport and solute clearance were low in the pig compared to humans and could be enhanced only to a limited extent by SAPD treatment. This was primarily due to the low peritoneal transport status of the pig (low to low‐average) compared to humans. In comparison, approximately 5% of PD patients are low‐transporters and 28% are low‐average transporters (Rumpsfeld, McDonald, & Johnson, 2006). MTAC of urea, creatinine, and phosphate were 3‐ to 5‐fold lower in the pig than in PD‐patients (Clerbaux, Francart, Wallemacq, Robert, & Goffin, 2006; Fischbach et al., 2004; Teixidó‐Planas, 2002). Consequently, solute concentrations in peritoneal effluent entering the SAPD system were low, accounting for the low absolute removal by the system. Of note, in vitro studies showed that in case of higher toxin delivery to the system the capacity of the system was sufficient to remove the daily toxin production (van Gelder, Ligabue, et al., 2020). The increase of the MTAC during SAPD treatment (1.1‐ to 1.3‐fold for urea and 1.2‐ to 2.3‐fold for creatinine vs. a static dwell) was lower than previously reported in patients treated with CFPD (1.5‐ to 2.5‐fold for urea and 1.4‐ to 2.5‐fold for creatinine) (Amerling et al., 2001; Cruz et al., 2001; Freida & Issad, 2003; Raaijmakers et al., 2011). This might also be related to the pig's low peritoneal membrane permeability as patients with higher transport status may benefit more from the continuous circulation of the dialysate (Edefonti et al., 1995; Flanigan, Lim, & Pflederer, 1993; Holtta, Ronnholm, & Holmberg, 2000; Rodriguez, 1998; Vychytil, Lilaj, Schneider, Hörl, & Haag‐Weber, 1999) than patients with low membrane permeability (Freida & Issad, 2003). Indeed, the increase in MTAC of creatinine and phosphate was more pronounced during peritonitis, when peritoneal transport status was higher. Another explanation for the relatively limited increase in clearance in our study as compared to CFPD in humans may be that SAPD applies a tidal flow rate *via* a single‐lumen catheter, whereas previous studies used two separate single‐lumen catheters (one for the inflow of fresh dialysate and one for outflow, respectively). The use of a separate in‐ and outflow catheter eliminates the dead volume (~50 ml with the current system), may reduce recirculation (~31% in our study), improve intraperitoneal fluid mixing and may optimize the dialysate flow along the peritoneal membrane and increase the effective membrane area, which are both crucial for increasing the MTAC (Gotch, 2001).

Two other initiatives are known in the field of CFPD with sorbent‐based dialysate regeneration. First, the automated wearable artificial kidney for PD (AWAK PD^TM^, <2 kg (Automated Wearable Artificial Kidney, 2020)) designed by AWAK Pte Ltd (Singapore and Burbank, CA) uses a modified REDY sorbent system, containing urease for enzymatic hydrolysis of urea, activated carbon, and ion exchangers for dialysate regeneration and, similar to SAPD, a single‐lumen PD catheter to perform CFPD using a tidal mode with rapid cycling of 250 ml of dialysate (mean effective dialysate flow rate 33 ml/min). The system is designed for 24‐hr use which requires three sorbent cartridges per day. In a first‐in‐human clinical trial, 14 PD patients were treated for ≥10.5 hr per day for up to three days (Al‐Hwiesh et al., 2019). Prior to the replacement of a cartridge, an exchange with 1–2 L of fresh dialysate was performed. Plasma concentrations of urea, creatinine, and phosphate decreased significantly by 29%, 31%, and 17%, respectively, indicating that clearance increased during treatment with AWAK PD as compared to conventional PD that was applied prior to and after AWAK PD treatment. However, plasma clearances and MTACs were not reported. The second initiative is the Carry Life System (CLS) for PD designed by Triomed AB (Lund, Sweden) which uses activated carbon and ion‐exchangers for dialysate regeneration and a second PD catheter for CFPD with continuous recirculation of dialysate. The system requires a new sorbent cartridge (<5 kg) every 4 hr. In a first‐in‐human clinical trial, five PD patients were treated for eight hours. A dialysate flow rate of 17 ml/min was applied and urea, creatinine, and phosphate clearances of 10.6 ± 1.9, 8.1 ± 2.1, and 6.4 ± 1.3 ml/min, respectively, were achieved during treatment, which is comparable to the clearances achieved during the night with APD (Bammens et al., 2003; Paniagua et al., 2002). Thus, findings from these two trials in PD patients show that CFPD using sorbent‐based dialysate regeneration can provide solute clearances that are at least comparable to conventional PD, also when applying CFPD *via* tidal mode with one single‐lumen PD catheter (AWAK PD). Both initiatives used dialysate flow rates that were relatively low. An important question remains whether higher clearances can be achieved with higher dialysate flow rates, as applied with our SAPD system. In contrast to AWAK PD and the CLS, our SAPD nighttime system lacks miniaturization (compared to conventional PD). However, our system is a low‐risk system as opposed to the more complex urease‐based sorbent system of AWAK PD which needs a cation exchanger to bind ammonium formed during enzymatic hydrolysis of urea. Also, the CLS contains a cation exchanger for the adsorption of potassium. In exchange for ammonium and/or potassium, the cation exchangers release sodium and hydrogen that must be neutralized to prevent the sodium loading of the patient and acidosis, respectively. In addition, the cation exchangers remove calcium and magnesium ions which need to be replenished, adding to the complexity of the system. Another feature of our SAPD system is that the dialysate reservoir (in combination with the increase in MTAC due to CFPD) is expected to provide clearance of other (not yet identified) “non‐bound” uremic waste solutes that approaches that of conventional PD.

Although the SAPD system does not comprise a cation exchanger, removal of a limited amount of potassium, calcium, and magnesium by the sorbents was observed in vivo and in vitro *via* binding to negatively charged phosphate that is bound to FeOOH. Calcium and magnesium removal can, therefore, not be prevented by the preloading of the sorbents. For the nighttime system, calcium removal was prevented by the relatively high calcium concentration in the dialysate reservoir (1.75 mmol/L). In theory, increasing magnesium concentration in the dialysate reservoir will also prevent magnesium removal, but for the current study (unmodified) commercially available dialysate (Physioneal 35: [Mg] 0.25 mmol/L) was selected for practical reasons. If necessary, magnesium concentration can be increased in the dialysate reservoir of the final system.

Treatment with the SAPD day‐ and nighttime systems resulted in net base release (sum of bicarbonate and lactate) into the pig of 17 and 21 mmol, respectively, coinciding with an increase in plasma bicarbonate concentration, decrease in plasma chloride concentration, and an increase in venous pH. However, the total base release was relatively low as compared to the average daily nonvolatile acid production in humans of ~70 mmol (Scialla, Asplin, & Dobre, 2017). In healthy patients, the elimination of this acid load is achieved by the urinary excretion of hydrogen ions, both as titratable acid and as ammonium (Rose & Post, 2001). ESKD leads to the retention of hydrogen ions. To buffer the retained acid and prevent metabolic acidosis in ESKD, a base load is delivered *via* the dialysate in conventional dialysis. Base release by the SAPD system observed in this study may be too low to prevent metabolic acidosis in patients. However, the pigs had metabolic alkalosis, which limited bicarbonate release. In addition, slow equilibration between plasma and intraperitoneal compartment in the pig limited lactate release. In vitro, we have demonstrated that cumulative base release by the nighttime system is sufficient (≥70 mmol) in case of metabolic acidosis (van Gelder, Ligabue, et al., 2020). Importantly, the capacity of the sorbents to release bicarbonate is highly concentration‐dependent, an important safety characteristic of the device. This concentration dependency implies that bicarbonate release is lower at higher plasma (and thus intraperitoneal) bicarbonate concentrations, and *vice versa*. Consequently, the risk of metabolic alkalosis and acidosis is attenuated. In addition, the effect on plasma bicarbonate concentrations can be predicted at the start of each dialysis session, allowing the modification of the dialysate prescription as required.

The ultrafiltration rate was very variable in the pig. Although very high initial intraperitoneal glucose concentrations were prevented during SAPD, no definite conclusions could be drawn with regard to the efficacy of ultrafiltration with SAPD. During SAPD, the sorbents are loaded with glucose until equilibration is achieved, after which the sorbents will start to release glucose. This principle maintains a rather constant glucose concentration in the effluent of the system and therewith a rather constant osmotic pressure for fluid removal throughout the entire treatment, eliminating the need for a separate glucose infusion system. Of note, the sorbents are not preloaded with glucose in order to prevent the formation of toxic glucose degradation products during sterilization. Lowering intraperitoneal glucose concentrations may prolong PD technique survival, since the exposure of the peritoneal membrane to very high glucose concentrations causes pathological changes of the peritoneal membrane and eventually ultrafiltration failure (Wu et al., 2012). In literature, adequate ultrafiltration has been observed at relatively low maximum intraperitoneal glucose concentrations in humans treated with CFPD. Triomed AB (Lund, Sweden) treated five patients for eight hours with the Carry Life® system for ultrafiltration, a portable ultrafiltration device that continuously cycles a small volume of peritoneal dialysate *via* the single‐lumen PD catheter, while adding glucose to the fluid that is returned to the patient. Intraperitoneal glucose concentration was maintained at ~1.3% resulting in a ~3‐fold higher UFR compared with CAPD (Johansson, Braide, & de Leon, 2018). In addition, Freida *et al*. observed a ~3.5‐fold increase in ultrafiltration volume per gram of absorbed glucose with CFPD compared with nocturnal intermittent PD in five patients (Freida & Issad, 2003), and Raaijmakers et al. found a 9‐fold higher UFR in six children treated with CFPD compared with conventional PD when using PD solutions with the same (initial) glucose concentration (Raaijmakers et al., 2011).

Treatment with the SAPD system was well tolerated and no serious adverse events occurred that could be related to SAPD treatment. Peritonitis (unintentional) was present in 13 out of 18 experiments. In all cases of peritonitis, a pathogenic micro‐organism and/or increased leukocyte and neutrophil count >50% were detected in peritoneal effluent of the overnight dwell that was taken prior to SAPD treatment, suggesting that peritonitis was not caused by SAPD treatment but was most likely due to poor hygienic conditions, inherent to pigsties.

This study has limitations. First, the animal model was characterized by a low peritoneal transport status and high plasma bicarbonate concentration, not representative of the human situation. This may account for the limited solute removal, the limited increase in plasma clearance, and MTAC as compared with a static dwell and low net base release. However, modeling based on in vitro data showed that the SAPD system efficiently removes small solutes in case of higher solute delivery to the system assuming a ~1.5‐ to 3.5‐fold increase in MTAC with CFPD in humans at a flow rate of ~100–200 ml/min (Amerling et al., 2001; Cruz et al., 2001; Freida & Issad, 2003; Gotch, 2001; Raaijmakers et al., 2011), and may well provide significantly higher solute clearances compared with conventional PD. In addition, sufficient base release was observed in vitro. Second, ELAR could not be determined during SAPD and SPA experiments. Thus, the ultrafiltration rate could not be calculated and solutes that were reabsorbed by lymphatic drainage were not accounted for in the calculation of total mass transport and MTAC. Last, a 4‐hr static dwell (SPA) was used as a control because this is a validated method for the calculation of MTAC, which was an important outcome measure as SAPD treatment is expected to improve plasma clearance primarily by increasing the MTAC. However, as most PD patients are treated by automated PD (APD), this might have underestimated the efficacy of the control treatment as frequent exchanges with shorter duration may enhance solute removal.

In conclusion, treatment with the SAPD system is safe and small solute clearance appeared to be enhanced as compared to a static dwell. Furthermore, the SAPD system has the potential to prolong technique survival by avoiding the need for very high initial glucose concentrations and to lower peritonitis rates by reducing the number of exchanges and (dis)connections of the peritoneal catheter. An important question remains whether higher clearances improve outcomes. Although the ADEMEX trial found that a limited increase (~35%) in creatinine clearance did not improve patient survival (Paniagua et al., 2002), much higher clearances, as may be achieved with CFPD, could improve patient outcome. Future studies in humans or animal species with higher peritoneal transport should elucidate whether our SAPD system enhances clearance to a clinically relevant extent as compared to conventional PD and prolongs technique survival.

## CONFLICT OF INTEREST

The authors declare no conflict of interest.

## AUTHORS' CONTRIBUTIONS

Conceptualization, M.K.v.G., F.S., J.A.J., K.G.F.G.; Methodology, M.K.v.G., F.S., K.G.F.G.; Formal analysis, M.K.v.G., F.S., K.G.F.G.; Investigation, M.K.v.G., J.C.d.V., F.S., A.S.M.; Resources, F.S., K.G.F.G.; Data curation, M.K.v.G.; Writing – original draft preparation, M.K.v.G., K.G.F.G.; Writing – review & editing, J.C.d.V., F.S., D.H.M.H., G.L., S.G., J.A.J., M.C.V., M.A.B.R., R.S., G.C., K.G.F.G.; Supervision, F.S., J.A.J., K.G.F.G.; Project administration, M.K.v.G., F.S., D.H.M.H., K.G.F.G.; Funding acquisition, F.S., J.A.J., K.G.F.G.

## ETHICAL STATEMENT

In vivo experiments were approved by the Animal Experiments Committee (Utrecht, The Netherlands) (AVD115002015226, NTS 2015226‐4) and performed in accordance with national guidelines for the care and handling of animals.

## Supporting information



Supplementary Material

## References

[phy214593-bib-0001] Aguirre, A. R. , & Abensur, H. (2011). Protective measures against ultrafiltration failure in peritoneal dialysis patients. Clinics (Sao Paulo), 66(12), 2151–2157. 10.1590/S1807-59322011001200023 22189743 PMC3226613

[phy214593-bib-0002] Al‐Hwiesh, A. A. K. A. , Abdul‐Rahman, I. S. , Al‐Audah, N. N. N. , Al‐Hwiesh, A. , Al‐Harbi, M. , Taha, A. , … Alzawad, N. A. (2019). Tidal peritoneal dialysis versus ultrafiltration in type 1 cardiorenal syndrome: A prospective randomized study. International Journal of Artificial Organs, 42(12), 684–694. 10.1177/0391398819860529 31303099

[phy214593-bib-0003] Amerling, R. , DeSimone, L. , Inciong‐Reyes, R. , Pangilinan, A. , Folden, T. , Ronco, C. , … Levin, N. (2001). Clinical experience with continuous flow and flow‐through peritoneal dialysis. Seminars in Dialysis, 14(5), 388–390. 10.1046/j.1525-139X.2001.00099.x 11679110

[phy214593-bib-0004] Automated Wearable Artificial Kidney (AWAK) .(2020). Available online (accessed on 24 February 2020). Retrieved from http://awak.com/product/

[phy214593-bib-0005] Bammens, B. , Evenepoel, P. , Verbeke, K. , & Vanrenterghem, Y. (2003). Removal of middle molecules and protein‐bound solutes by peritoneal dialysis and relation with uremic symptoms. Kidney International, 64(6), 2238–2243. 10.1046/j.1523-1755.2003.00310.x 14633148

[phy214593-bib-0006] Clerbaux, G. , Francart, J. , Wallemacq, P. , Robert, A. , & Goffin, E. (2006). Evaluation of peritoneal transport properties at onset of peritoneal dialysis and longitudinal follow‐up. Nephrology Dialysis Transplantation, 21(4), 1032–1039. 10.1093/ndt/gfi344 16364990

[phy214593-bib-0007] Cruz, C. , Melendez, A. , Gotch, F. A. , Folden, T. , Crawford, T. L. , & Diaz‐Buxo, J. A. (2001). Single‐pass continuous flow peritoneal dialysis using two catheters. Seminars in Dialysis, 14(5), 391–394. 10.1046/j.1525-139X.2001.00098.x 11679111

[phy214593-bib-0008] Davenport, A. (2009). Peritonitis remains the major clinical complication of peritoneal dialysis: The London, UK, peritonitis audit 2002–2003. Peritoneal Dialysis International: Journal of the International Society for Peritoneal Dialysis, 29(3), 297–302. 10.1177/089686080902900314 19458302

[phy214593-bib-0009] de Fijter, C. W. , Oe, P. L. , Nauta, J. J. , van der Meulen, J. , ter Wee, P. M. , Snoek, F. J. , & Donker, A. J. (1991). A prospective, randomized study comparing the peritonitis incidence of CAPD and Y‐connector (CAPD‐Y) with continuous cyclic peritoneal dialysis (CCPD). Advances in Peritoneal Dialysis, 7, 186–189. 1680422

[phy214593-bib-0010] Diaz‐Buxo, J. A. , Cruz, C. , & Gotch, F. A. (2000). Advances in end‐stage renal diseases 2000. Continuous‐flow peritoneal dialysis. Preliminary results. Blood Purification, 18(4), 361–365. 10.1159/000014463 10965082

[phy214593-bib-0011] Edefonti, A. , Consalvo, G. , Picca, M. , Giani, M. , Damiani, B. , Ghio, L. , & Galato, R. (1995). Dialysis delivery in children on nightly intermittent and tidal peritoneal dialysis. Pediatric Nephrology, 9(3), 329–332. 10.1007/BF02254202 7632525

[phy214593-bib-0012] Eknoyan, G. , Beck, G. J. , Cheung, A. K. , Daugirdas, J. T. , Greene, T. , Kusek, J. W. , … Toto, R. (2002). Effect of dialysis dose and membrane flux in maintenance hemodialysis. New England Journal of Medicine, 347(25), 2010–2019. 10.1056/NEJMoa021583 12490682

[phy214593-bib-0013] Fischbach, M. , Terzic, J. , Chauve, S. , Laugel, V. , Muller, A. , & Haraldsson, B. (2004). Effect of peritoneal dialysis fluid composition on peritoneal area available for exchange in children. Nephrology Dialysis Transplantation, 19(4), 925–932. 10.1093/ndt/gfg518 15031351

[phy214593-bib-0014] Flanigan, M. J. , Lim, V. S. , & Pflederer, T. A. (1993). Tidal peritoneal dialysis: Kinetics and protein balance. American Journal of Kidney Diseases, 22(5), 700–707. 10.1016/S0272-6386(12)80433-1 8238016

[phy214593-bib-0015] Freida, P. , & Issad, B. (2003). Continuous flow peritoneal dialysis: Assessment of fluid and solute removal in a high‐flow model of "fresh dialysate single pass". Peritoneal Dialysis International, 23(4), 348–355. 12968842

[phy214593-bib-0016] Fresenius Medical Care . (2018). Annual report 2018. Available online (accessed on 24 February 2020). Retrieved from https://www.fresenius.com/media/FME_Annual‐Report_2018.pdf

[phy214593-bib-0017] Gotch, F. A. (2001). Kinetic modeling of continuous flow peritoneal dialysis. Seminars in Dialysis, 14(5), 378–383. 10.1046/j.1525-139X.2001.00096.x 11679108

[phy214593-bib-0018] Holtta, T. , Ronnholm, K. , Holmberg, C. (2000). Adequacy of dialysis with tidal and continuous cycling peritoneal dialysis in children. Nephrology Dialysis Transplantation, 15, 1438–1442. 10.1093/ndt/15.9.1438 10978404

[phy214593-bib-0019] Johansson, A. C. , Braide, M. , de Leon, C. et al (2018). Peritoneal ultrafiltration with a stable glucose concentration using the Carry Life® UF System [Abstract]. Nephrology Dialysis Transplantation, 33, SP529.

[phy214593-bib-0020] Krediet, R. T. , Lindholm, B. , & Rippe, B. (2000). Pathophysiology of peritoneal membrane failure. Peritoneal Dialysis International: Journal of the International Society for Peritoneal Dialysis, 20(Suppl 4), S22–S42. 10.1177/089686080002004S03 11098927

[phy214593-bib-0021] Mujais, S. , & Story, K. (2006). Peritoneal dialysis in the US: Evaluation of outcomes in contemporary cohorts. Kidney International, 70, S21–S26. 10.1038/sj.ki.5001912 17080107

[phy214593-bib-0022] Paniagua, R. , Amato, D. , Vonesh, E. , Correa‐Rotter, R. , Ramos, A. , Moran, J. , … Mexican Nephrology Collaborative Study Group . (2002). Effects of increased peritoneal clearances on mortality rates in peritoneal dialysis: ADEMEX, a prospective, randomized, controlled trial. Journal of the American Society of Nephrology, 13(5), 1307–1320. 11961019 10.1681/ASN.V1351307

[phy214593-bib-0023] Raaijmakers, R. , Schroder, C. H. , Gajjar, P. et al (2011). Continuous flow peritoneal dialysis: First experience in children with acute renal failure. Clinical Journal of the American Society of Nephrology, 6(2), 311–318. 10.2215/CJN.00330110 21030578 PMC3052221

[phy214593-bib-0024] Roberts, M. B. C. , & Zaragosa, J. S. (2016). Pig trial of automated wearable artificial kidneys based on peritoneal dialysis [Abstract]. Expert Review of Medical Devices, 15, 323–336.

[phy214593-bib-0025] Rodriguez, A. M. (1998). Automated peritoneal dialysis: A Spanish multicentre study. Nephrology Dialysis Transplantation, 13, 2335–2340. 10.1093/ndt/13.9.2335 9761518

[phy214593-bib-0026] Rose, B. D. , & Post, T. W. (2001). Clinical physiology of acid‐base and electrolyte disorders (5th ed., p. 328). New York: McGraw‐Hill.

[phy214593-bib-0027] Rumpsfeld, M. , McDonald, S. P. , & Johnson, D. W. (2006). Higher peritoneal transport status is associated with higher mortality and technique failure in the Australian and New Zealand peritoneal dialysis patient populations. Journal of the American Society of Nephrology, 17(1), 271–278. 10.1681/ASN.2005050566 16306167

[phy214593-bib-0028] Scialla, J. J. , Asplin, J. , Dobre, M. et al (2017). Higher net acid excretion is associated with a lower risk of kidney disease progression in patients with diabetes. Kidney International, 91(1), 204–215. 10.1016/j.kint.2016.09.012 27914710 PMC5518613

[phy214593-bib-0029] Shinaberger, J. H. , Shear, L. , & Barry, K. G. (1965). Increasing efficiency of peritoneal dialysis: Experience with peritoneal‐extracorporeal recirculation dialysis. Transactions ‐ American Society for Artificial Internal Organs, 11, 76–82. 10.1097/00002480-196504000-00015 14329117

[phy214593-bib-0030] Smit, W. , Langedijk, M. J. , Schouten, N. , Van Den Berg, N. , Struijk, D. G. , & Krediet, R. T. (2000). A comparison between 1.36% and 3.86% glucose dialysis solution for the assessment of peritoneal membrane function. Peritoneal Dialysis International: Journal of the International Society for Peritoneal Dialysis, 20(6), 734–741. 10.1177/089686080002000626 11216568

[phy214593-bib-0031] Teixidó‐Planas, J. (2002). Peritoneal function and adequacy calculations: Current programs versus PD Adequest 2.0. Peritoneal Dialysis International: Journal of the International Society for Peritoneal Dialysis, 22(3), 386–393. 10.1177/089686080202200314 12227398

[phy214593-bib-0032] van Gelder, M. K. , Jong, J. A. W. , Folkertsma, L. , Guo, Y. , Blüchel, C. , Verhaar, M. C. , … Gerritsen, K. G. F. (2020). Urea removal strategies for dialysate regeneration in a wearable artificial kidney. Biomaterials, 234, 119735 10.1016/j.biomaterials.2019.119735 31958714

[phy214593-bib-0033] van Gelder, M. K. , Ligabue, G. , Giovanella, S. , Bianchini, E. , Simonis, F. , Hazenbrink, D. H. M. , … Gerritsen, K. G. F. (2020). In vitro efficacy and safety of a system for sorbent assisted peritoneal dialysis. American Journal of Physiology‐Renal Physiology, 319(2), F162–F170. 10.1152/ajprenal.00079.2020 32475132

[phy214593-bib-0034] van Gelder, M. K. , Simonis, F. , Monninkof, A. M. , Hazenbrink, D. H. M. , Bajo Rubio, M. A. , Selgas, R. , … Gerritsen, K. G. F. (2019). Evaluation of a Wearable Artificial Kidney for Peritoneal Dialysis in a Uremic Pig Model [Abstract], ASN Washington.

[phy214593-bib-0035] Vychytil, A. , Lilaj, T. , Schneider, B. , Hörl, W. H. , & Haag‐Weber, M. (1999). Tidal peritoneal dialysis for home‐treated patients: Should it be preferred? American Journal of Kidney Diseases, 33, 334–343. 10.1016/S0272-6386(99)70309-4 10023647

[phy214593-bib-0036] Waniewski, J. , Werynski, A. , Heimburger, O. , & Lindholm, B. (1991). Simple models for description of small solute transport in peritoneal dialysis. Blood Purification, 9(3), 129–141. 10.1159/000170009 1801855

[phy214593-bib-0037] Wu, H. Y. , Hung, K. Y. , Huang, T. M. , Hu, F.‐C. , Peng, Y.‐S. , Huang, J.‐W. , … Wu, K.‐D. (2012). Safety issues of long‐term glucose load in patients on peritoneal dialysis–a 7‐year cohort study. PLoS One, 7(1), e30337 10.1371/journal.pone.0030337 22303440 PMC3264614

