# Supplementary material for: Evaluation of a system for sorbent‐assisted peritoneal dialysis in a uremic pig model
Source: Physiol Rep. 2020 Dec 5;8(23):e14593. doi: 10.14814/phy2.14593 (PMC7718839; doi:10.14814/phy2.14593)
Supplement: Supplementary file 1 — Supplementary Material [file PHY2-8-e14593-s001.docx]

**Supplemental Figures**


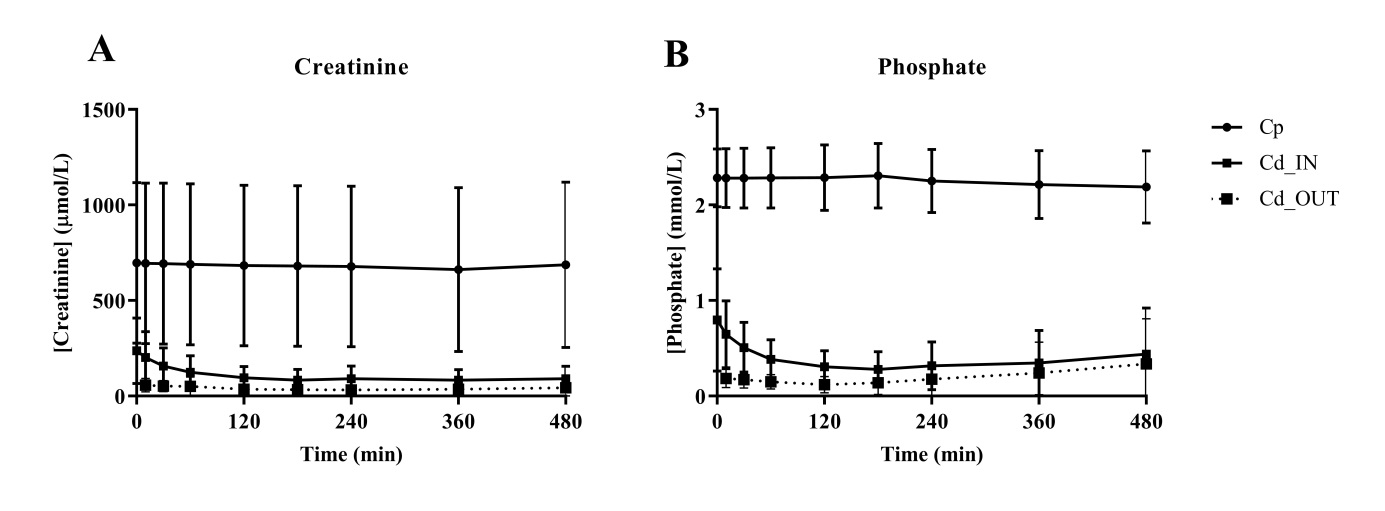


Figure S1. Mean (±SD) plasma (Cp) and dialysate creatinine (A) and phosphate (B) concentrations in the SAPD in- (Cd_IN) and outgoing (Cd_OUT) line during experiments with the SAPD day- (n=3 experiments in n=1 pig) and nighttime system (n=15 experiments in n=3 pigs).


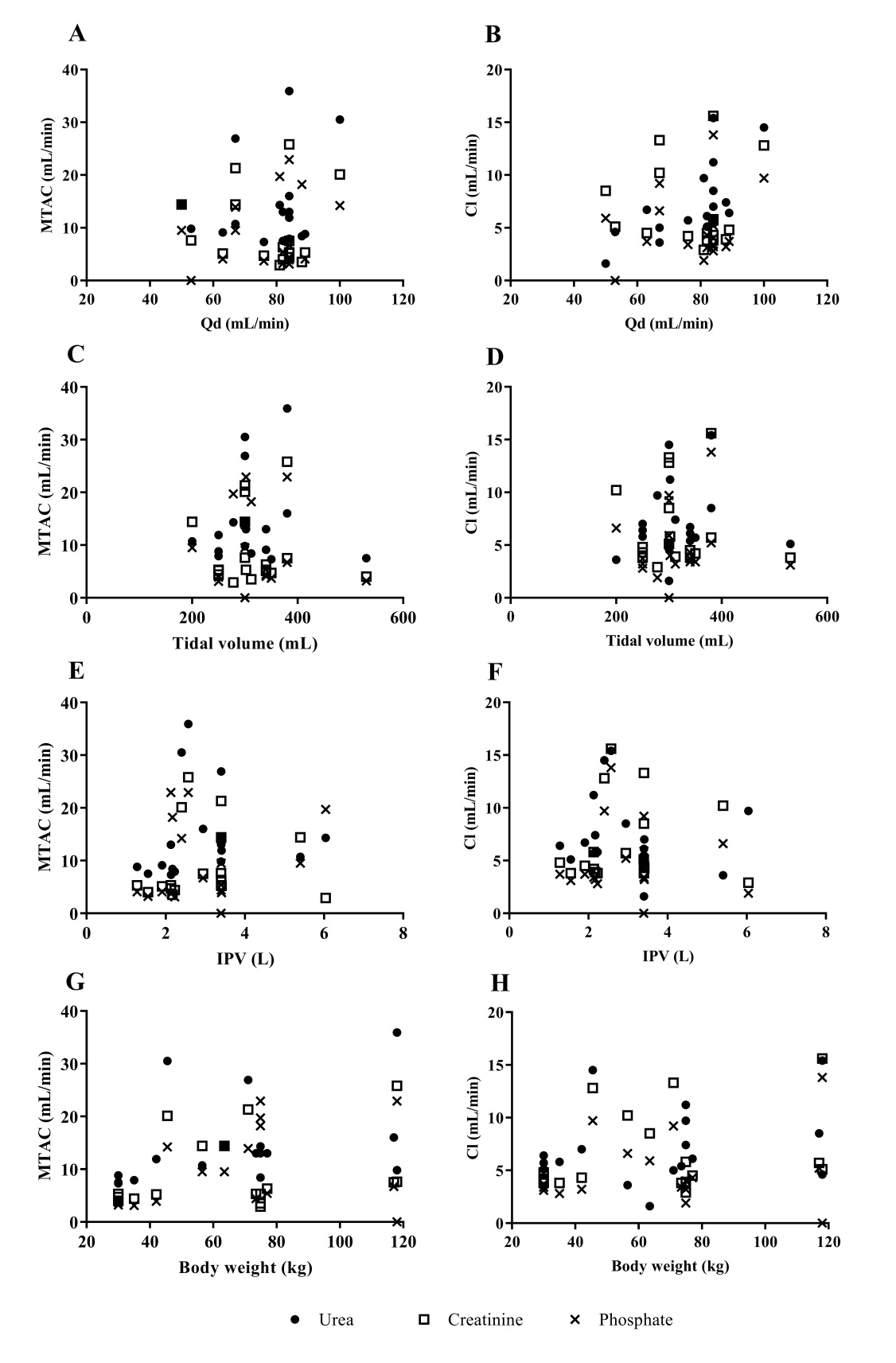


Figure S2. The association between the mean effective dialysate flow rate (Qd, A, B), tidal volume (C, D), intraperitoneal volume (IPV, E, F), bodyweight (G, H), and the mass transfer area coefficient (MTAC) and plasma clearance (Cl) of urea, creatinine and phosphate during SAPD experiments. Each data point represents the MTAC or Cl of a solute during a single experiment. n=15 experiments were performed with the nighttime system in n=3 pigs (n=7, n=5 and n=3 per pig), n=3 experiments were performed with the daytime system in n=1 pig.

**Supplemental Tables**

Table S1. Ultrafiltration during SAPD and SPA experiments.

|  | **[glucose] in 10-L reservoir and filling** | **UFR (mL/min)** | **Max. IP [glucose] (%)** | **Mean IP [glucose] (%)** |
| --- | --- | --- | --- | --- |
| SAPD nighttime system | 1.36% (n=3) | 0.13 ± 0.15 | 0.96 ± 0.15 | 0.78 ± 0.05* |
|  | 1.72% (n=5) | -0.47 ± 0.71 | 0.99 ± 0.15 | 0.84 ± 0.13 |
|  | 2.27% (n=2) | 1.06; 0.71 | 1.22; 1.31 | 1.09; 1.26 |
| SPA | 1.36% (n=19) | 0.19 ± 0.89 | 1.20 ± 0.10 | 0.90 ± 0.08 |
|  | 2.27% (n=2) | 1.00; 0.80 | 2.07; 1.96 | 1.38; 1.38 |

**Mean ± standard deviation is presented. In case of n=2, the results per experiment are presented separated by a semicolon. *P = 0.04 (**Student’s paired t-test)**.** IP, intraperitoneal; SPA, standard peritoneal permeability analysis; UFR, ultrafiltration rate. Ultrafiltration volume was estimated based on intraperitoneal volume at the start and end of the experiment, corrected for total sampling volume. Since effective lymphatic adsorption rate could not be determined, the ultrafiltration data presented here underestimate ‘true’ UFR values.

Table S2. Mean dialysate-to-plasma concentration ratios at 4 h of urea, creatinine, phosphate and potassium during SAPD and SPA experiments.

|  | **Peritonitis** | **SPA**  **(n=28)** | **SAPD daytime (n=3)** | **SAPD daytime *vs* SPA** | ***P**** | **SAPD nighttime (n=15)** | **SAPD nighttime *vs* SPA** | ***P**** |
| --- | --- | --- | --- | --- | --- | --- | --- | --- |
| D/P urea | NO | 0.66 ± 0.09 |  |  |  | 0.35 ± 0.07 | ×0.5 | **0.006** |
|  | YES | 0.77 ± 0.10 | 0.86 ± 0.06 | ×1.1 | 0.365^§^ | 0.40 ± 0.13 | ×0.5 | **<0.001** |
| D/P creatinine | NO | 0.40 ± 0.05 |  |  |  | 0.08 ± 0.00 | ×0.2 | **<0.001** |
|  | YES | 0.56 ± 0.14 | 0.26 ± 0.02 | ×0.5 | **0.002** | 0.15 ± 0.10 | ×0.3 | **<0.001** |
| D/P phosphate | NO | 0.33 ± 0.05 |  |  |  | 0.06 ± 0.00 | ×0.2 | **<0.001** |
|  | YES | 0.49 ± 0.14 | 0.27 ± 0.01 | ×0.5 | **0.002** | 0.14 ± 0.11 | ×0.3 | **<0.001** |
| D/P potassium | NO | 0.88 ± 0.01 |  |  |  | 0.14 ± 0.11 | ×0.6 | **<0.001** |
|  | YES | 0.86 ± 0.04 | 0.92 ± 0.02 | ×1.1 | 0.093^§^ | 0.57 ± 0.10 | ×0.7 | **<0.001** |

D/P, dialysate-to-plasma concentration ratio at 4 h.*P-value was calculated using a Student’s paired t-test for differences between consecutive SAPD and SPA experiments. Significant differences are shown in bold font. §The SAPD daytime system does not comprise a urea removal system or cation exchanger for removal of potassium. n=15 experiments were performed with the nighttime system in n=3 pigs (n=7, n=5 and n=3 per pig), n=3 experiments were performed with the daytime system in n=1 pig and 28 SPA experiments were performed in n=3 pigs (n=8, n=16 and n=4 per pig).
